# Diagnostic challenges of rarely well-differentiated adenocarcinoma of the stomach

**DOI:** 10.3389/pore.2025.1612163

**Published:** 2025-07-07

**Authors:** Tian Qin, Yong Wang, Zebin Xiao, Lili Ma, Chao Fan, Chongyu Zhu, Luqiao Luo, Qingling Zhang, Chao Liu

**Affiliations:** ^1^ Department of Pathology, Guangdong Provincial People’s Hospital (Guangdong Academy of Medical Sciences) Southern Medical University, Guangzhou, China; ^2^ Department of Pathology, Affiliated Hospital of Guizhou Medical University, Guiyang, Guizhou, China; ^3^ Department of Hepatobiliary Surgery, Guangzhou Eighth People’s Hospital, Guangzhou Medical University, Guangzhou, China; ^4^ Department of Pathology, Heyou Hospital, Foshan, China

**Keywords:** stomach neoplasms, gastric adenocarcinoma of the fundic gland, diagnosis, welldifferentiated, MUC6

## Abstract

**Background:**

Fundic gland tumors are a rare subtype of gastric tumors with fundic gland differentiation. This group of tumors has a low incidence rate and shows indistinctive cellular atypia, obvious structural atypia, special tissue morphology, and clinical prognosis, thus leading to diagnostic challenges.

**Aim:**

We aimed to investigate the clinical and endoscopic characteristics and pathological features of gastric adenocarcinoma of the fundic gland (GA-FG) to provide a better understanding of this disease.

**Methods:**

We collected data from patients diagnosed as having GA-FG at Guangdong Provincial People’s Hospital between January 2019 and April 2024. The analysis focused on their clinical data, endoscopic characteristics, pathological morphological characteristics, immunohistochemistry results, treatment, and prognosis.

**Results:**

Among the four patients were two men and two women (age range, 52–65 years). The tumors were mainly located in the gastric fundus and gastric body, and the lesions commonly had a superficial bulge. Three patients had an initial diagnosis of oxyntic gland adenoma, which was diagnosed as GA-FG after complete resection. These tumors were negative for MUC5AC, but showed diffuse strong positivity for MUC6 and pepsinogen I, and synaptophysin expression.

**Conclusion:**

GA-FG is a rare gastric tumor with unique morphological features. As it is difficult to diagnose with a biopsy, immunohistochemistry plays an important role in the differential diagnosis. Oxyntic gland adenoma can be regarded as the intramucosal stage of GA-FG. Although all patients were negative for MUC5AC expression, MUC6 and pepsinogen I can help the diagnosis of GA-FG.

## Introduction

Fundic gland tumors are a rare subtype of gastric tumors with fundic gland differentiation, and they originate from chief cells or parietal cell precursor cells of the mucosal layer. In recent years, these have been reported to mainly include oxyntic gland adenoma (OGA), a newly proposed type of gastric adenocarcinoma of the fundic gland (GA-FG) and gastric adenocarcinoma of the fundic gland mucosa type (GA-FGM) [1]. The differentiation of GA-FG into chief cells was first reported by Tsukamoto et al. in 2007 [2]. Subsequently, Ueyama et al. summarized 10 cases in 2010 and officially named this phenomenon GA-FG [3]. The fifth edition of the World Health Organization’s (WHO) classification of tumors of the digestive system divides gastric oxyntic gland-type tumors into OGA and GA-FG [4]. Both are tumors derived from the fundic glands, with small atypia and high similarity to non-tumor fundic gland cells. Latest research classifies oxyntic gland adenoma (OGA) and GA-FG as low-grade lesions which are more likely to be accompanied by submucosal infiltration (about 60%). OGA with submucosal infiltration is diagnosed as GA-FG. GA-FG can be divided into three types, which include the chief cell predominant type, parietal cell predominant type, and mixed phenotype. Among these, chief cell predominant type is the most prevalent histological type, accounting for approximately 99% of all GA-FG cases [4]. Currently, this group of tumors is widely believed to have a low incidence rate, small cellular atypia, obvious structural atypia, and special tissue morphology and clinical prognosis. Some scholars believe that the genetic pathway of GA-FG may be different from that of conventional gastric adenocarcinoma, and may originate from the chief cell line and parietal cell line. The *H. pylori* infection rate on the basis of reports thus far is <40%, and it may also be related to the use of proton pump inhibitors [5]. However, diagnosis is made challenging by the lack of overall understanding and systematic reviews. GA-FGs are rarely observed clinically, and most cases are reported in Asia (China, South Korea, and Japan) [6]. Owing to the eradication treatment of *Helicobacter pylori* and improvements in public health, the number of digestive endoscopies has gradually increased, which has led to a gradual increase in related reports on GA-FGs in recent years. However, although GA-FG was first described in 2007, research on this topic has not gained momentum since then, which has led to the existing literature on GA-FG being limited.

In this study, we report four cases of GA-FG. The diagnoses were not derived from the initial gastroscopy findings but instead relied on postoperative pathology reports. Most of them were initially diagnosed as oxyntic gland adenomas before secondary examination. Fundic adenocarcinoma was the final diagnosis after extensive surgical resection. Herein, we summarized the clinical characteristics, endoscopic morphology, and pathomorphological characteristics of these cases and discussed the diagnostic key points and mechanisms of tumors derived from the fundic gland; we believe our findings will improve the understanding of this disease among clinicians and pathologists. These cases were followed up, and the current treatment results were satisfactory, which encouraged us to share our findings through this report.

## Materials and methods

We reviewed the GA-FG pathology with archives at Guangdong Provincial People’s Hospital between January 2019 and April 2024, and a total of four patients who were pathologically diagnosed with GA-FG were included in this study. Available information on clinical details (including age, sex, and medication history), macroscopic appearance, microscopic features of the lesion and the adjacent mucosa, and immunohistochemical data were also collected. The EnVision two-step method was used for immunohistochemical staining, and the slices were incubated with primary antibodies (MUC5AC, MUC6, MUC2, P53, pepsinogen I, synaptophysin (Syn), chromogranin A (CgA), CD56, desmin, and Ki-67). Hematoxylin staining was performed, observed under the microscope, and analyzed. Some information was not available as several consultations were referrals from other doctors or hospitals, which explains the missing information. Histopathological examination assessed the architectural patterns, the presence of cytonuclear atypia (i.e., nuclear enlargement, presence of nucleoli, and hyperchromasia), the presence of necrosis and stromal changes, the depth of involvement, and immunohistochemical staining. The architectural patterns observed included clustered or solid glands with or without well-defined lumina, anastomosing cords, dilated glands with or without folds, complex glands with multiple layers of cells, cribriform glands. All procedures performed in this study involving human participants were in accordance with the ethical standards of the institutional and/or national research committee and complied with the 1964 Helsinki Declaration and its later amendments or comparable ethical standards. All patients agreed to and signed the informed consent form for the purpose of publication. The analysis focused on their clinical data, endoscopic characteristics, morphological and pathological characteristics, immunohistochemistry results, treatment, and prognosis.

## Results

### Clinical and endoscopic characteristics

**FIGURE 1:**
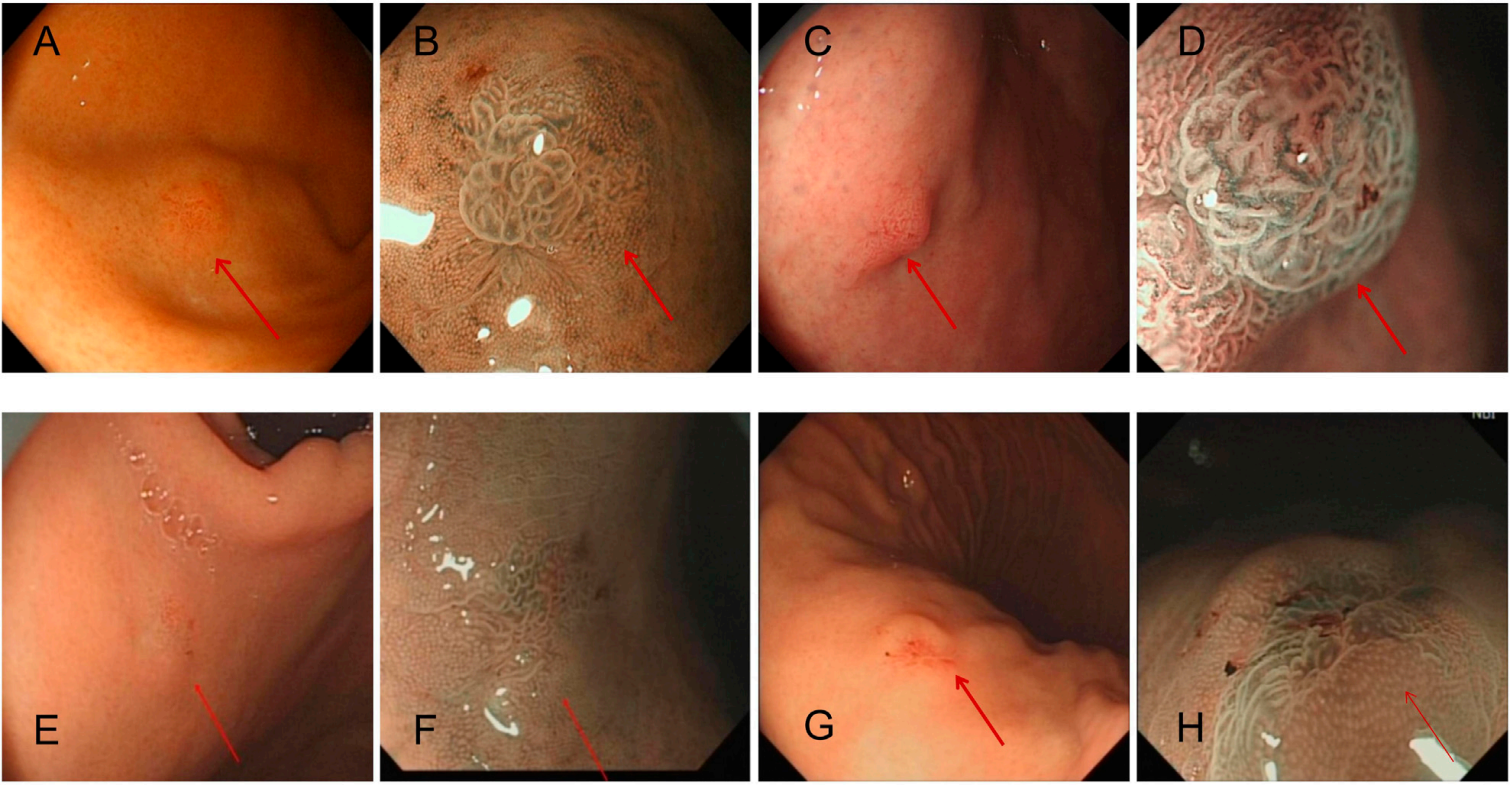
Each patient’s representative endoscopic images of GA-FG (arrows), which appears as a small swelling lesion in fundus of stomach in endoscopic images. Magnified narrow band imaging **(B,D,F,H)** revealed a mound-like protrusion, with a red, rough surface and faintly visible branching vessels. **(A,B)**: Case 1 with an elevated tumor of 6 mm in diameter in the non-atrophic mucosa of the gastric fundus [**(A)**, white-light image; **(B)**, NBI-ME]. **(C,D)**: Case 2 with an elevated tumor of 7 mm in diameter in the non-atrophic mucosa of the gastric fundus [**(C)**, white-light image; **(D)**, NBI-ME]. **(E,F)**: Case 3 with an elevated tumor of 5 mm in diameter in the non-atrophic mucosa of the gastric fundus [**(E)**, white-light image; **(F)**, NBI-ME]. Finally, **(G,H)**: Case 4 with an elevated tumor of 6 mm in diameter in the non-atrophic mucosa of the gastric body [**(G)**, white-light image; **(H)**, NBI-ME].

Among the four patients were two men aged 52 and 65 years and two women aged 52 and 64 years; the overall median age was 58 years, and the average age was 58.25 years. The clinical symptoms were abdominal pain, epigastric discomfort, and hunger pangs (one case each) in the three patients who came to the hospital for treatment, and one case was discovered by physical examination. Endoscopic features revealed that the tumors were mainly located in the fundus of the stomach in three cases, whereas in one case, there were in the body of the stomach. According to the Paris classification, the lesions were identified as superficial raised type (type 0-IIa) in three cases and superficial depressed type (type 0-IIa + IIc) in one case. The surface mucosa was red in three cases and faded in one case. Narrow band imaging combined with magnifying endoscopy revealed that the interfossa part of the apical gland was widened, abnormal blood vessels were exposed, and the glands were arranged in a disorderly manner and were partially fused in all cases (Figure 1). The tumor diameter ranged from 0.5 to 0.7 cm, with an average tumor diameter of 0.6 cm. All four cases underwent endoscopic submucosal dissection (ESD). Postoperative telephonic follow-up was conducted for 4–28 months, and the last follow-up was on 1 May 2024 (Table 1). Postoperative follow-up was carried out every 3 months for the first 2 years and every 6 months thereafter. Follow-up results showed that their survival status was good, and there was no disease progression, lymph node metastasis, or distant metastasis at the last postoperative follow-up.

**TABLE 1:** Clinical presentation, endoscopy, and follow-up time of the four patients.

Patient	Age (years)	Tumor size	Location in stomach	*Helicobacter pylori* infection	Macroscopic NBI-ME	Macroscopic features	MVP	Follow-up (months)
1	52	0.6 cm × 0.6 cm	Fundus	Negative	Single	Type 0-IIa	Irregular	4
2	64	0.7 cm × 0.6 cm	Fundus	Negative	Single	Type 0-IIa	Irregular	46
3	65	0.5 cm × 0.5 cm	Fundus	Negative	Single	Type 0-IIa + IIc	Irregular	15
4	52	0.6 cm × 0.5 cm	Gastric body	Negative	Single	Type 0-IIa	Irregular	13

^a^ NBI-ME: narrow band imaging-magnifying endoscopy, MVP: microvascular pattern.

### Pathomorphologic features

**FIGURE 2:**
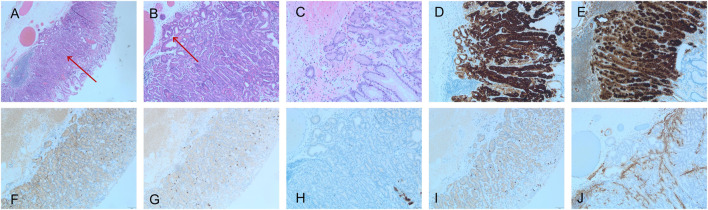
GA-FG pathology, **(A–C)** exhibited HE staining, while **(D–J)** displayed IHC staining. **(A)** Low magnification images of gastric adenocarcinoma of the fundic gland-type (GA-FG) (HE, ×40) showing the tumor (red arrow), which blends imperceptibly with normal oxyntic glands (left and right portions of the image). Note the submucosal invasion lacks any desmoplasia or myxoid change. **(B)** The complex glandular architecture seen at a higher magnification revealing an anastomosing and so-called “endless glands” pattern (HE, ×100). Note occasional cystic glands in the lesion that may mimic a fundic gland polyp (red arrow). **(C)** Higher power of GA-FG showing the tumor cell atypia is not obvious. (HE, ×100). **(D)** MUC6 (×100) and **(E)** pepsinogen I showed diffuse positive staining (×100). **(F)** Syn was positive (weak) (×40); **(G)** CgA was negative. (×40); **(H)** MUC5AC was negative (×100), and **(I)** showed that Ki-67 does not exhibit focal concentration (×40). **(J)** Desmin immunostaining (×100) revealed the breach of the muscularis mucosae by neoplastic glands invading into the superficial submucosa.

All four cases showed differentiation into chief cells, with most areas of the tumor surface covered with normal foveolar epithelium. The tumor cells comprised well-differentiated chief cells. The cells were columnar and slightly enlarged, had mild nuclear atypia, and were located at the base. We also observed increased density of tightly packed fundus glands, mildly basophilic cytoplasm, mild nuclear atypia, rare mitotic figures, upward movement of the nuclei from the basal layer, glandular hyperplasia, and irregular anastomosis between glands; these features collectively formed a complex and disordered “endless glandular” structure, and no necrosis was seen. These characteristics seemed to denote a transition between the tumor and the surrounding glands. In all four cases, the tumors infiltrated the submucosal layer, with an invasion depth of 0.1–0.2 mm, none of the tumors invaded the submucosa deeper than 500 μm (SM1). The infiltration was limited to the mucosa, and the stromal reaction was not obvious (Figure 2). In all four cases, there were no obvious mitotic figures, no vascular invasion, and no *H. pylori* infection, atrophy, or intestinal metaplasia in the mucosa surrounding the tumor.

### Immunohistochemical features

In four patients, tumor cells showed diffuse strong positivity for MUC6, but normal gastric epithelial cells were negative. In addition, tumor cells were negative for MUC5AC, whereas normal gastric epithelial cells were distinctly positive for it. In addition, all patients were diffuse strong positive for pepsinogen I. Meanwhile, the expression pattern of CD56 was positive but intensity unstable, and in terms of Syn expression, the patients showed completely negative to diffuse strong positive expression pattern. All patients were negative for CgA expression, and their Ki-67 was <5% (Table 2; Figure 2).

**TABLE 2:** Pathological and immunohistochemical features.

Patient	Depth of invasion (µm)	First diagnosis	Final diagnosis	MUC5AC	MUC6	MUC2	P53	Pepsinogen Ⅰ	Syn	CgA	CD56	Ki-67
1	100	GA-FA	GA-FG	(−)	(+++)	NA	(−)	(+++)	(−)/(++)	(−)	NA	(<1%+)
2	100	OGA	GA-FG	(−)	(+++)	(−)	(−)	(+++)	Partly (+- ++)	(−)	Partly (++–+++)	5%
3	200	OGA	GA-FG	(−)	(+++)	(−)	NA	(+++)	(+++)	(−)	Weak (+)	(<1%+)
4	200	OGA	GA-FG	(−)	(+++)	(−)	(−)	(+++)	(+)	(−)	NA	(<1%+)

^a^ OGA: oxyntic gland adenoma, GA-FG: gastric adenocarcinoma of the fundic gland, Syn: synaptophysin, CgA: chromogranin A, NA: not applicable.

## Discussion

GA-FG is a rare gastric tumor subtype originating from chief cells or parietal cell precursor cells of the mucosal lamina. Its morphological features are similar to those of typical oxyntic gland tumors and immunohistochemical staining markers are similar to those of OGA. The 2019 WHO classification of tumors of the digestive system proposed defining OGA and GA-FG based on the presence or absence of submucosal invasion and emphasized that over 60% of OGA can progress to GA-FG [4]. Ueyama et al. [4] suggest that there is a morphological continuum from OGA to GA-FG and believe that OGA lesions with histological characteristics similar to GA-FG should be regarded as the intramucosal stage of GA-FG. Both OGA and GA-FG belong to the same disease. However, other experts hold a controversial opinion that OGA is a mucosal prolapse ectopia.

The age of onset of GA-FG in our patients was 52–65 years (average age, 58.25 years), which is consistent with the age of onset of 60–70 years indicated by the WHO classification of tumors of the digestive system. There was no special gender bias in terms of incidence; however, due to the small sample size of our study, it is impossible to make an accurate judgment. GA-FG mostly affects the fundus of the stomach. In our study, GA-FG affected the fundus of the stomach in three patients and the body of the stomach in one patient. However, GA-FG can reportedly also be found in ectopic fundic glands. Manabe et al. [7] and Uozumi et al. [8] found GA-FG in fundic glands with duodenal ectopia. Although all of our patients showed solitary lesions, GA-FG can reportedly occur as multiple lesions [9]. It can also occur simultaneously with other malignant gastric tumors, such as gastric signet ring cell carcinoma [10, 11] and neuroendocrine neoplasms [12]. Furthermore, it has also been reported to manifest as a submucosal lesion in autoimmune gastritis [13].

In our patients, endoscopy findings revealed that the maximum diameter of GA-FG was 0.5–0.7 cm (average diameter, 0.6 cm). Based on endoscopic gross morphology, three cases were classified as Paris classification 0–0-IIa, and one case was classified as Paris classification 0-IIa + IIc. All tumors showed dilated small blood vessels, rough surface, and visible dendritic blood vessels. The lesions appeared to be flat and raised with or without central depression; they often had a red tone, which may fade partially, but the tone identified herein was slightly different from the previously reported faded tone of GA-FG [14]. Furthermore, previous reports have stated the presence of brownish pigmentation as well [15]. The following were the observed characteristics of OGA and GA-FG under narrow band imaging-magnifying endoscopy (NBI-ME) [16].: (1) lack of a clear demarcation line; (2) increased crypt opening; (3) widening of the intervening part; and (4) regular microsurface pattern and microvascular pattern on the surface. The possibility of fundic gland-type adenocarcinoma needs to be considered if the following findings are noted during endoscopy: (i) the presence of a submucosal bulge in the lesion, (ii) no atrophy of the surrounding gastric mucosa, (iii) lesion location in the fundus of the stomach, (iv) the absence of infection, and (v)the color of the gastric fundus lesion was observed to fade along its periphery. [17].

Among our four patient specimens, three were initially diagnosed as OGA and were later diagnosed as GA-FG after complete resection by endoscopic submucosal dissection (ESD) surgery. Owing to the presence of submucosal infiltration, the remaining one case was diagnosed as GA-FG at the initial diagnosis itself. For a more accurate classification, some scholars suggest that the clinical ESD or endoscopic mucosal resection (EMR) resection be further performed depending on the biopsy-based diagnosis in the oxyntic gland-type tumor; if infiltration of the submucosal layer is observed, the recommended diagnosis is GA-FG [18], and we also suggested that desmin staining should be used to mark the muscularis mucosae to better identify if the tumor had invaded beyond the muscularis mucosae. Given its feasibility, safety, and efficacy, ESD earns its recommendation as a treatment approach for GA-FG [19].

Light microscopy revealed specific morphological characteristics of GA-FG. Notably, tumor cells mainly comprised highly differentiated columnar cells, imitating oxyntic gland cells (mainly chief cells). Furthermore, they appeared to have a light gray-blue color, a basophilic cytoplasm, and slightly swollen nuclei. Moreover, in some lesions, tumor cells were noted to have a coarsely granular eosinophilic cytoplasm similar to that of parietal cells. The surface of the lesion was covered by normal gastric epithelium, whereas deeper areas of the tumor showed irregular branching and expansion. The tumors are arranged in irregular arrangement, complex adenoid, forming an “endless gland” pattern. Some studies have reported new morphological findings wherein they observed other morphological patterns, such as solid glands with or without a well-defined lumen and dilated glands with invaginations, and the complex glands have multiple layers of cells and cribriform glands [20]. These clustered solid glands and anastomotic cords for which immunohistochemistry showed weak to moderate synaptophysin and/or CD56 positivity may have been misidentified as neuroendocrine tumors [20]. Among our four patients, all were positive for synaptophysin and two were positive for CD56. However, all were negative for CgA, and this general absence of CgA excludes a major neuroendocrine origin, particularly as most foregut neuroendocrine tumors are typically positive for this marker. Notably, synaptophysin and CD56 are not specific markers for neuroendocrine differentiation, particularly in the foregut. The cause of the lesions associated with this spectrum is uncertain [20]. In all cases, MUC6 and pepsinogen I staining were strongly positive, whereas MUC5AC staining was negative, and Ki-67/MIB1 marker indices were all <5%, we believe that this ratio is very low; the positive cells are distributed irregularly, and no proliferative zone is formed.

The current molecular mechanisms of GA-FGs and gastric fundic gland-derived tumors mainly focus on Wnt/β-catenin, GNAS/KRAS mutation, and ERK 1/2 MAPK and Shh signaling pathways. Some studies believe that the occurrence of gastric fundus gland-derived tumors is related to the mutation of the CTNNB1 and AXIN genes and the nuclear aggregation of β-catenin protein in the cells, which leads to the activation of the Wnt/β-catenin pathway. A disturbed Wnt/β-catenin pathway can affect gastrin and thus promote the occurrence of gastric fundus gland tumors [21]. However, β-catenin intranuclear aggregation on IHC staining was rarely observed in previous studies [20, 22]. Nomura et al. [23] using NGS testing found that there were multiple genetic alterations in gastric fundus gland-derived tumors, including mutations in GNAS, KRAS, PIK3CA, and CDKN2A and co-mutations in GNAS and CDKN2A. Most of these genes were related to the MAPK pathway, suggesting that tumor occurrence may be related to the activation of the MAPK pathway. Upon comparing the incidence of GNAS mutations in OGA and GA-FG, their frequency was found to be 33.3% and 14.8%, respectively [1]. Relevant studies have shown that activating mutations in GNAS are able to promote tumorigenesis through the activation of the Wnt/β-catenin pathway or the ERK 1/2 MAPK pathway. GNAS mutations have also been detected in GA-FG samples of the Chinese population [24]. In addition, some studies have shown that the Shh signaling pathway-related proteins in gastric fundus gland-derived tumors are independent of the Wnt/β-catenin pathway and play a crucial role in maintaining fundus gland cell proliferation and differentiation, and furthermore, the Shh protein has different expression patterns between OGA and GA-FG [25]. Recently, related studies have suggested new mechanisms showing the relevance to the occurrence and development of GA-FG, including ectopic expression and normal transactivation ability of NKX2-1/TTF-1, suggesting that NKX2-1 plays an essential role in GA-FG development [26]. Moreover, the diffuse MIST1 expression and decreased α-1,4-linked N-acetylglucosamine (αGlcNAc) glycosylation of MUC6 are the obvious markers of gastric neoplasms with oxyntic gland differentiation, suggesting that decreased αGlcNAc glycosylation of MUC6 and diffuse positivity of MIST1 also play an important role in tumor origin [27]. In addition, the decreased level of SP70 is a feature specific to fundic gland neoplasms, including OGAs and GA-FGs. Therefore, SP70 can serve as a potential biomarker in the identification and differential diagnosis of fundic gland neoplasms [28]. In addition, some reports show that GA-FG rarely can show microsatellite instability. However, a patient diagnosed with GA-FG was found to have the AXIN2 mutation instead of the GNAS mutation. This patient also showed microsatellite instability, with the tumor lesion invading the subserous layer with lymphatic vessel infiltration [29]. Moreover, this patient with no GNAS mutation but carrying an AXIN2 frameshift mutation showed distant metastasis and had a poor prognosis [29]. In conclusion, the molecular mechanisms underlying gastric fundus gland-derived tumors are unclear and may involve a process in which multi-gene, multi-signaling pathways are involved.

In 2021, Ueyama et al. [1] presented the concept of gastric epithelial tumors with fundic gland mucosal differentiation [gland mucosa lineage: gastric epithelial neoplasms of fundic gland mucosa lineage (GEN-FGML)] and improved the classification. GEN-FGML was classified into three types: OGA, GA-FG, and GA-FGM. OGA and GA-FG had very low risk of recurrence and progression and were classified as low-grade fundic gland tumors. GA-FG was further classified into the main cell type (99%), mural cell type, and mixed type. However, GA-FGM was categorized as a high-grade tumor because of the aggressive ability. Besides gastric fundus gland differentiation, GA-FGM shows minor concave epithelium and/or mucous gland differentiation. A diagnosis of GA-FG needs to be differentiated from not only GA-FGM but also other benign and malignant tumors of the stomach, such as gastric gland polyps, gastric concave adenoma, pyloric adenoma, “crawling type adenocarcinoma,” and neuroendocrine tumors. Among all the classifications of gastric fundus gland-derived tumors, the one by Ueyama et al. is the most complete so far, and it reflects the biological behavior of different histological types, which has been applied in clinical practice. The malignancy grades reported by Ueyama et al. are important not only to analyze carcinogenesis but also in clinical use to determine whether additional surgical resection is required after endoscopic therapy. According to the 2018 Japanese Guidelines for the Treatment of Gastric Cancer (5th edition) [30], additional surgical resection is required when the gastric cancer depth of submucosal invasion is equal to or greater than 500 µm. However, GA-FG exhibits little vascular invasion without recurrence or metastasis regardless of the depth of submucosal infiltration, and such GA-FG cases may not require additional expanded surgical resection.

Advanced GA-FG can reportedly present with abnormal clinicopathological features [31]. Although GA-FG cases may transform into high-grade malignancy during tumor progression, Ueyama et al. insist that there is no clear evidence that GA-FG can progress into GA-FGM by maintaining GNAS mutations, and reversion of the differentiation status to acquiring multilineage differentiation potential.

This study has some limitations associated with the retrospective nature of the study and the small number of enrolled patients. Furthermore, we did not assess the relevancy of β-catenin associated with GA-FG. Mutation of the CTNNB1 and AXIN genes and the nuclear aggregation of β-catenin protein lead to the activation of the Wnt/β-catenin pathway. A disturbed Wnt/β-catenin pathway can affect gastrin and thus promote the occurrence of gastric fundus gland tumors. However, previous studies indicate that β-catenin is not detected in the nucleus during immunohistochemical analysis. There are still many questions about the asynchrony of activation of the nuclear β-catenin pathway and protein expression, which may be a direction for further research Although additional GNAS mutations (42.9%) have been reported, the patients in our study were not tested for those, this is a limitation of the study.

GA-FG is a rare gastric tumor with unique morphological features; as it is difficult to diagnose with a biopsy, immunohistochemistry plays an important role in the differential diagnosis. OGA can be regarded as the intramucosal stage of GA-FG. Although all patients were negative for MUC5AC expression, MUC6 and pepsinogen I can help the diagnosis of gastric fundus gland-derived tumors. The lesions were completely removed by ESD and did not require further surgical resection. Immunohistochemical staining should be performed by a pathologist to confirm the diagnosis if GA-FG is suspected on endoscopy. The long-term prognosis is good for most patients. The etiology and pathogenesis of GA-FG deserve more attention as it differs from conventional gastric adenocarcinoma.

## Data Availability

The raw data supporting the conclusions of this article will be made available by the authors, without undue reservation.
